# Porcine Epidemic Diarrhea Virus Infection Subverts Arsenite-Induced Stress Granules Formation

**DOI:** 10.3389/fmicb.2022.931922

**Published:** 2022-07-04

**Authors:** Xiaozhen Guo, Kejian Yu, Zhonghao Xin, Liping Liu, Yuehua Gao, Feng Hu, Xiuli Ma, Kexiang Yu, Yufeng Li, Bing Huang, Zhengui Yan, Jiaqiang Wu

**Affiliations:** ^1^Shandong Key Laboratory of Immunity and Diagnosis of Poultry Diseases, Institute of Poultry Science, Shandong Academy of Agricultural Sciences, Jinan, China; ^2^Shandong Provincial Key Laboratory of Animal Biotechnology and Disease Control and Prevention, College of Animal Science and Technology, Shandong Agricultural University, Taian, China; ^3^Shandong Key Laboratory of Disease Control and Breeding, Institute of Animal Science and Veterinary Medicine, Shandong Academy of Agricultural Science, Jinan, China; ^4^Shandong Key Laboratory of Animal Resistant Biology, College of Life Sciences, Shandong Normal University, Jinan, China

**Keywords:** PEDV replication, stress granules, sodium arsenite, papain-like protease, interaction mechanism

## Abstract

Stress granules (SGs) are dynamic cytoplasmic protein-RNA structures that form in response to various stress conditions, including viral infection. Porcine epidemic diarrhea virus (PEDV) variant-related diarrhea has caused devastating economic losses to the swine industry worldwide. In this study, we found that the percentage of PEDV-infected cells containing SGs is nearly 20%; meanwhile, PEDV-infected cells were resistant to sodium arsenite (SA)-induced SGs formation, as demonstrated by the recruitment of SGs marker proteins, including G3BP1 and TIA1. Moreover, the formation of SGs induced by SA treatment was suppressed by PEDV papain-like protease confirmed by confocal microscopy. Further study showed that PEDV infection disrupted SGs formation by downregulating G3BP1 expression. Additionally, PEDV replication was significantly enhanced when SGs' assembly was impaired by silencing G3BP1. Taken together, our findings attempt to illuminate the specific interaction mechanism between SGs and PEDV, which will help us to elucidate the pathogenesis of PEDV infection in the near future.

## Introduction

Porcine epidemic diarrhea (PED) caused by the porcine epidemic diarrhea virus (PEDV) variant is characterized by acute enteric infection and high mortality in suckling piglets and occasional endemics, leading to significant economic losses to the global swine industry (Lee, 2015; Niederwerder and Hesse, 2018). PEDV is an enveloped, single-stranded positive-sense RNA virus belonging to the genus Alphacoronavirus in the family Coronaviridae of the order Nidovirales (Li et al., 2012). The viral genome is ~28 kb and encodes at least seven open reading frames (ORFs). The two largest ORFs (ORF1a and ORF1b) located in the two-thirds of the genome downstream of the 5′UTR are further processed into 16 non-structural proteins, nsp1 to nsp16. The other one-third genome encodes the structural and accessory proteins including spike (S) glycoprotein, small envelope (E) protein, membrane (M) protein, nucleocapsid (N) protein, and accessory protein (ORF3). A better understanding of the interactions between PEDV and host might contribute to the development of more effective control measures (Guo et al., 2017).

Stress granules (SGs) are dynamic cytoplasmic protein-RNA structures that form in response to various stress conditions including viral infection (Anderson and Kedersha, 2009). The major components of SGs are untranslated mRNAs, mainly including Ras GTPase-activating protein-binding protein 1 (G3BP1), T-cell internal antigen 1 (TIA1), eukaryotic translation initiation factors (eIF), and TIA1-related protein (TIAR) (Panas et al., 2016). G3BP1 and TIA-1 are usually used as SGs markers to identify SGs formation. Accumulating evidence has shown that SGs play an important role in regulating viral replication. For example, several viruses, such as a respiratory syncytial virus (RSV), have been reported to benefit from SGs formation (Lindquist et al., 2010). However, SGs are also considered as an indication of an antiviral innate response to many viruses (Onomoto et al., 2014; Yoneyama et al., 2016). Many viruses have evolved strategies to overcome the antiviral effect of SGs by degrading or sequestering its key components, such as G3BP1 or TIA-1/TIAR to prevent the formation of SGs (Humoud et al., 2016; Le Sage et al., 2017). It was documented that Infectious Bronchitis Virus (IBV) antagonized the formation of SGs by nsp15 *via* reducing the viral dsRNA accumulation and sequestering/depleting the critical component of SGs (Gao et al., 2021). Foot-and-mouth disease virus (FMDV) leader protease cleaves G3BP1 and G3BP2 to inhibit SG formation (Visser et al., 2019). A previous study reported that PEDV infection induced caspase-8 mediated G3BP1 cleavage to subvert SG formation (Sun et al., 2021). However, in addition to the above-mentioned mechanisms, whether there is another mechanism to inhibit SG formation during PEDV infection remains largely unknown.

In the present study, we demonstrated that PEDV infection disrupted SA-induced SGs formation *via* downregulating G3BP1 expression. Further study indicated that PEDV papain-like protease could subvert SGs formation. Moreover, impairment of SG formation dramatically enhanced PEDV replication. Collectively, this study will lay the foundation for further investigation of PEDV infection and SG formation from the perspective of the protein encoded by the virus itself.

## Materials and Methods

### Cells and Viruses

The kidney cell lines of the African green monkey, Vero-E6 cells, were cultured and maintained in Dulbecco's modified Eagle's medium (DMEM), supplemented with 10% fetal bovine serum (Invitrogen, Carlsbad, CA, USA), 100 U/mL penicillin, and 100 μg/mL streptomycin sulfate in a humidified 37°C, 5% CO_2_ incubator. The PEDV variant strain SDSX16 (Accession no. MH117940.1) was isolated from a suckling piglet with acute diarrhea in our lab.

### Virus Infection

Vero cells were cultured for nearly 24 h for 80% confluence and washed twice with serum-free medium. Then, the cells were mock-infected or infected with PEDV at a multiplicity of infection (MOI) of 0.1 and incubated with serum-free DMEM containing 8 μg/mL trypsin (Invitrogen) for 12 h, and then were treated with 5 mM SA for 30 min.

### Plasmid Construction

cDNA encoding PEDV PLP1, PLP2, and nsp5 were amplified by RT-PCR from total RNA of PEDV using specific primers (Table 1), and the three genes were cloned into pCAGGS-HA vector, respectively. All constructs were validated by DNA sequencing.

**Table 1:** Primer sequences used in this experiment.

**Primer name**	**Primer sequence**
PEDV-PLP1-F	CCCATCGATATCTCACAGGATCTGCT
PEDV-PLP1-R	ACCCCCGGGTCAAGCAGCATCATAAAAGTT
PEDV-PLP2-F	CAAGAATTCGTATCCACACCTGATGAT
PEDV-PLP2-R	CCCGGTACCCTACTCTGAGACGACAACATTT
PEDV-NSP5-F	AAAGAATTCGCTGGCTTGCGTAAGATGGC
PEDV-NSP5-R	CCCGCATGCTTACTGAAGATTAACGCCATACATTTGA
PEDV-M-F	CGTACAGGTAAGTCAATTAC
PEDV-M-R	GATGAAGCATTGACTGAA
β-actin-F	TTAGTTGCGTTACACCCTTTC
β-actin-R	ACCTTCACCGTTCCAGTT

### Reagent, siRNA, and Cell Transfection

Sodium arsenite (SA) was obtained from Sigma-Aldrich (St. Louis, MO, USA) and used at a concentration of 5 mM. The siRNAs targeting monkey G3BP1 gene or negative control siRNA were purchased from GenePharma and used at a concentration of 50 nM using Lipofectamine 3000 reagent (Invitrogen) according to the manufacturer's instructions. The siRNA sequences used in this study are listed in Table 2.

**Table 2:** siRNA sequences used in this experiment.

**Gene name**	**siRNA sequence(5^**′**^-3^**′**^)**	**siRNA sequence(3^**′**^-5^**′**^)**
G3BP1	UCAACAUGGCGAAUCUUGGUGUGGC	GCCACACCAAGAUUCGCCAUGUUGA
NC	CAAGAUGCAGCAGUAUGUAUGUGAA	UUCACAUACAUACUGCUGCAUCUUG

### Antibody

Rabbit poly antibodies (pAbs) against TIA1, G3BP1, eIF3B, mouse monoclonal antibody (mAb) against β-actin, horseradish peroxidase (HRP)-conjugated goat anti-rabbit, and HRP-conjugated goat anti-mouse IgG were all purchased from ABclonal Technology Co., Ltd. (Wuhan, China). Mouse mAb against HA-tag was purchased from Medical & Biological Laboratories Co., Ltd (MBL). Mouse mAb against PEDV S protein was generated by our laboratory. Alexa Fluor 488-conjugated goat anti-mouse IgG and Fluor Cy5-conjugated goat anti-rabbit IgG were obtained from Servicebio Biotechnology Co., Ltd. (Wuhan, China).

### Confocal Fluorescence Microscopy

Vero cells were seeded on coverslips and transfected with PCAGGS-HA associated expression plasmids or G3BP1 siRNA, or were infected with PEDV alone for the indicated time points. The cells were then fixed with cold 4% paraformaldehyde for 10 min. After permeabilization and blocking, the cells were then incubated with mouse mAbs directed against the HA-tag or S protein, or rabbit pAbs against TIA1, G3BP1, and eIF3B, respectively for 1 h, and were then inoculated with Alexa Fluor 488-conjugated goat anti-mouse IgG or Fluor Cy5-conjugated goat anti-rabbit IgG antibody for 30 min. Cell nuclei were counterstained with 0.01% 4′,6-diamidino-2-phenylindole (DAPI, Invitrogen). The fluorescent images were examined under a confocal laser scanning microscope (LSM 510 Meta, Carl Zeiss, Munich, Germany).

### Western Blot Assay

The PEDV-infected and mock-infected cells were harvested and lysed at 12 hpi. The protein concentration was quantified by the BCA protein assay kit, and equal amounts of protein samples from each sample were mixed with 5 × sample loading buffer and boiled for 10 min and then separated by 12% sodium dodecyl sulfate polyacrylamide gel electrophoresis (SDS-PAGE). The proteins were electro-transferred to 0.45 μm PVDF membranes (Millipore, Mississauga, ON, Canada). Membranes were blocked with 5% (w/v) skim milk-TBST at room temperature for 2 h and then incubated overnight at 4°C with primary antibody to G3BP1 and mouse mAb against β-actin, respectively. The membranes were washed with TBST and then incubated with horseradish peroxidase (HRP) conjugated goat anti-rabbit IgG or goat anti-mouse IgG (ABclonal, Wuhan, China) at 37°C for 1 h. The protein bands were visualized using the Clarity™ Western ECL Blotting Substrate (Bio-Rad, Hercules, CA, USA). The protein blots were quantified by Image J software (National Institutes of Health, Bethesda, MD, USA).

### Quantitative Real-Time PCR

Total RNA was extracted from PEDV-infected or mock-infected cells using Simply P Total RNA Extraction Kit (BioFlux, Europe) according to the manufacturer's protocol. A total of 1 μg RNA from each sample was subsequently reverse-transcribed to cDNA by using oligo (dT) as the primer (Roche, Mannheim, Germany). The relative quantitative real-time PCR was performed in an Applied Biosystems ViiA 7 real-time PCR system (Applied Biosystems, Foster City, CA, USA) as previously described (Guo et al., 2017). The primers are listed in Table 1.

### Quantification of SGs Formation

For the quantification of SGs formation, more than 10 high-powered fields (HPFs) were randomly obtained for each sample. PEDV-S protein was used to detect PEDV infection. HA-tag was used to monitor the effective expression of PLP1, PLP2, and nsp5, respectively. The number of SG-positive cells was counted by the results of staining for the presence of G3BP1, TIA1, or eIF3B proteins. The relative percentage of cells with SGs was calculated by: cells positive with G3BP1, TIA1, or eIF3B individually, and PEDV-S or HA tag divided by cells positive with PEDV-S or HA tag ×100% (Zhou et al., 2017; Gao et al., 2021).

### Statistical Analysis

Data of three independent experiments were expressed as the means with SEM. The student's *t*-test was applied for the statistical analyses. A *p* < 0.05 was considered as statistically significant.

## Results

### PEDV Infection Does Not Trigger SGs Formation in Most Infected Cells

**Figure 1:**
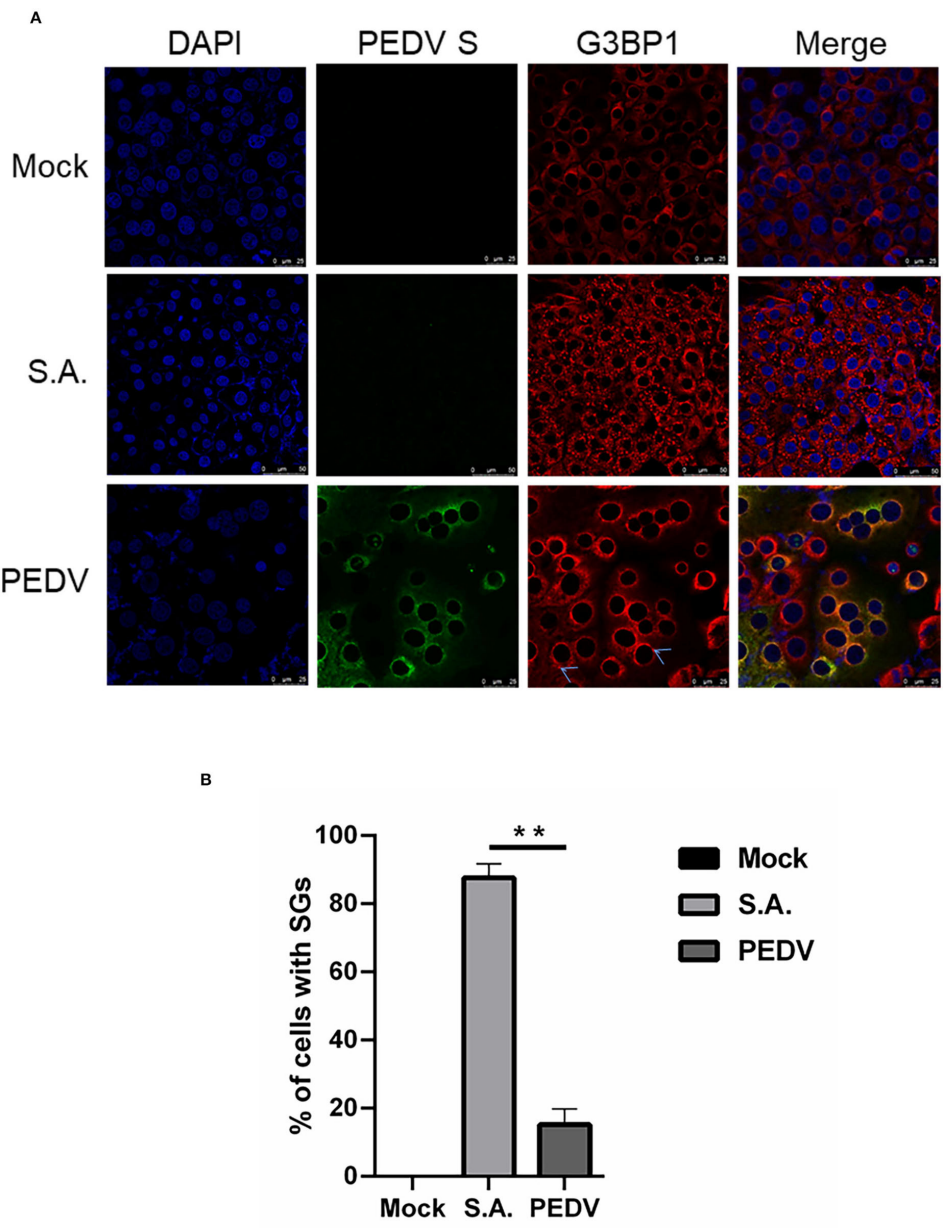
PEDV infection prevents SGs formation in most infected cells. **(A)** Vero cells were mock-infected or infected with PEDV for 12 h. Vero cells treated with 5 mM SA for 30 min served as the positive control. Then the cells were fixed and analyzed by confocal microscopy. Mouse mAb specific for PEDV S was used to detect PEDV infection (green). Rabbit pAb specific for G3BP1 (red) was used to detect SGs. Nuclei were stained with DAPI (blue). **(B)** The number of cells infected with PEDV and cells containing SGs were counted. The relative percentage of cells with SGs was calculated by: cells positive with G3BP1 and PEDV-S divided by cells positive with PEDV-S × 100%. The data were presented as mean ± SEM of three independent experiments (*t*-test, **p < 0.01).

The immunofluorescence analysis of G3BP1 was performed to detect SGs formation. A monoclonal antibody specific for PEDV-S protein was used to detect PEDV infection. The SGs were examined at 12 h after PEDV infection. Cells treated with SA, a potent SG-inducer, served as the positive control. Meanwhile, the mock-infected cells were regarded as the negative control. Unsurprisingly, the percentage of SGs positive cells reached more than 80% in SA-treated cells, while no obvious SGs were observed in mock-infected cells (Figure 1A). The percentage of PEDV-infected cells containing SGs is nearly 20% (Figure 1B), which is consistent with a previous report (Gao et al., 2021). These data suggest that PEDV infection cannot effectively trigger SGs formation in most infected Vero cells.

### PEDV-Infected Cells Are Resistant to SA Induced SGs Formation

**Figure 2:**
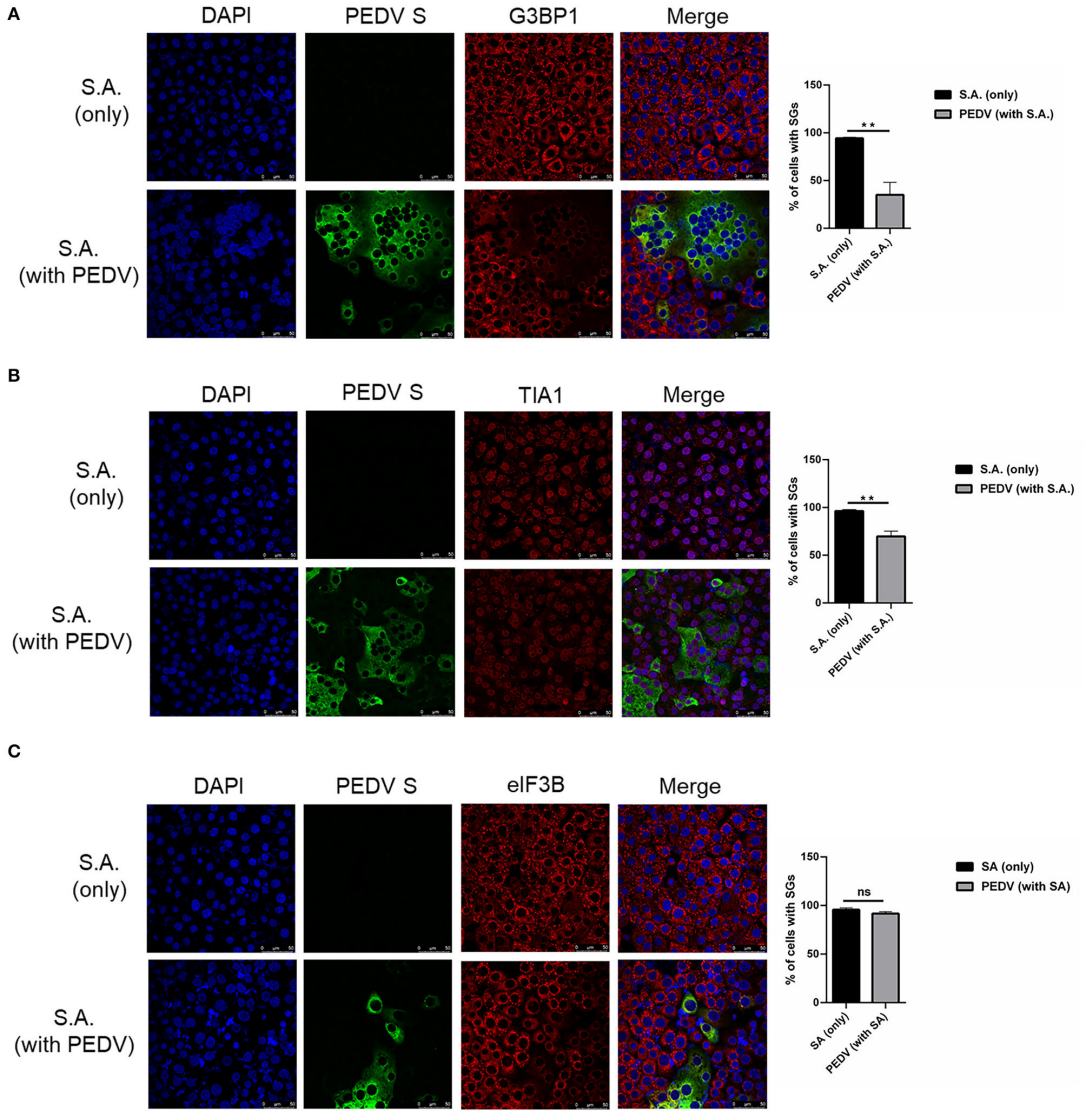
PEDV replication inhibits SGs formation induced by SA treatment. **(A–C)** Vero cells were mock-infected or infected with PEDV for 12 h and then treated with SA for 30 min. Cells were fixed and analyzed by confocal microscopy. Rabbit pAb specific for G3BP1 (**A**: red), TIA1 (**B**: red) or eIF3B (**C**: red) were used to detect SGs. Nuclei were stained with DAPI (blue). The percentage of SGs-positive cells detected by G3BP1, TIA1 or eIF3B was quantified. Error bars show standard deviations. The data were presented as mean ± SEM of three independent experiments (*t*-test, **p < 0.01).

To investigate whether PEDV-infection interferes with the formation of SGs induced by SA treatment, Vero cells were infected with PEDV for 12 h and then treated with SA for 30 min prior to immunofluorescence staining. As expected, SGs appeared in non-infected cells treated with SA, whereas PEDV-infected cells showed fewer SGs, and no specific SGs were detected by G3BP1 in syncytial cells. The percentage of cells with SGs was above 90% in mock-treated cells after SA treatment, while the percentage of SGs was below 40% in PEDV-infected cells (Figure 2A). In addition, the SGs' positive rate is close to 100% when only treated with SA and decreased to about 60% after PEDV infection detected by TIA1 (Figure 2B). Surprisingly, SGs formation treatment with SA detected by eIF3B was not influenced by PEDV infection (Figure 2C). In conclusion, it was proposed that PEDV infection could induce SGs, and PEDV-infected cells are resistant to SA-induced SGs formation.

### PEDV Papain-Like Protease Participate in Suppressing SGs Formation

**Figure 3:**
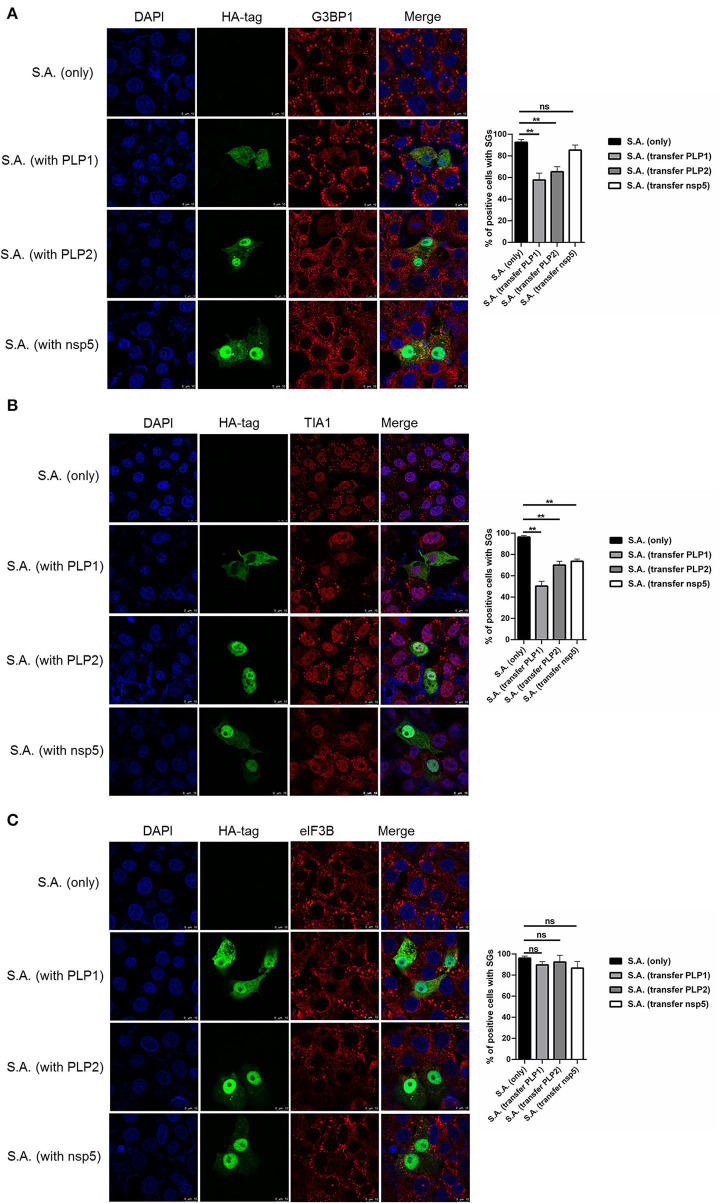
PEDV papain-like protease participate in suppressing SGs formation. **(A–C)** Vero cells were respectively transfected with an expression vector containing PLP1, PLP2,or nsp5 for 24 h and then treated with 5mM SA for 30 min. Rabbit pAb specific for G3BP1 (**A**: red), TIA1 (**B**: red), or eIF3B (**C**: red) was used to detect SGs. Mouse mAb specific for HA-tag was used to detect protein expression. Nuclei were stained with DAPI (blue). The percentage of SG-positive cells detected by G3BP1, TIA1, or eIF3B was quantified. Error bars show standard deviations. The data were presented as mean ± SEM of three independent experiments (*t*-test, **p < 0.01).

To investigate how PEDV-infected cells are resistant to SA-induced SGs formation, PEDV protease PLP1, PLP2, and nsp5 were successfully expressed in Vero cells. We found that PEDV PLP1 or PLP2, which have L protease activities, suppressed the SGs formation induced by SA treatment, as demonstrated by the recruitment of SGs marker proteins, including G3BP1 and TIA1. Conversely, PEDV nsp5 which has 3C-like protease activities showed remarkable effect on SGs formation indicated by TIA1, not G3BP1 (Figures 3A,B). Meanwhile, we found that not only PEDV PLP1 and PLP2 but also nsp5 has inconspicuous effect on SGs detected by eIF3B (Figure 3C). These results indicated that PEDV proteases PLP1 and PLP2 play an important part in suppressing SA-induced SGs.

### PEDV Infection Disrupted SGs Formation by Downregulating G3BP1 Expression

**Figure 4:**
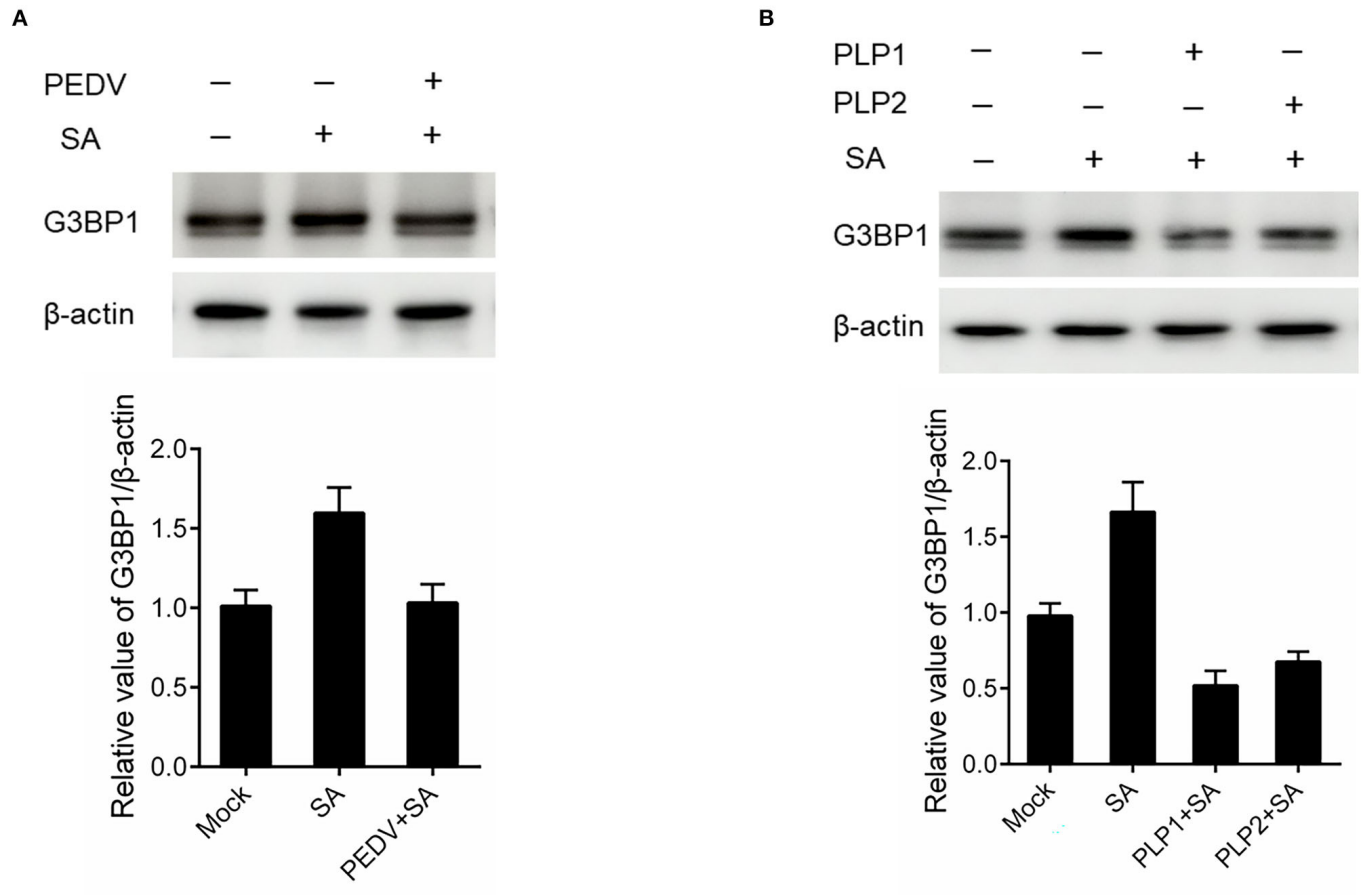
PEDV infection disrupted SGs formation by downregulating G3BP1 expression. **(A)** Vero cells were infected with PEDV for 12 h and treated with SA for 30 min. **(B)** Vero cells were respectively transfected with an expression vector containing PLP1 or PLP2 for 24 h and then treated with 5 mM SA for 30 min. The level of G3BP1 was analyzed by western blot. The expression of β-actin served as a control. The relative value of G3BP1/β-actin was analyzed by using Image J Software.

It is well known that G3BP1 is proposed to be key for the nucleation of SG assembly. As shown in Figure 2A, a loss of G3BP1 has been found in PEDV-infected cells treatment with SA than non-infected. These data led us to further investigate whether PEDV infection could downregulate G3BP1 expression by western blot. As shown in Figure 4A, the content of G3BP1 was increased in mock-infected cells treatment with SA compared with no treatment, while the protein level of G3BP1 was decreased in PEDV-infected cells treatment with SA. Meanwhile, we found that PEDV papain-like protease could suppress SA-induced SGs formation. Next, we determined the expression of G3BP1 in cells transfected with PEDV papain-like protease. As expected, the expression of G3BP1 was also decreased in PLP1 or PLP2-containing cells treatment with SA compared with cells lacking PLP1 or PLP2 (Figure 4B). It was speculated that PEDV infection might subvert SGs formation by downregulating the expression of G3BP1, which might depend on PEDV papain-like protease.

### Silencing G3BP1 Expression Had a Positive Influence on PEDV Replication

**Figure 5:**
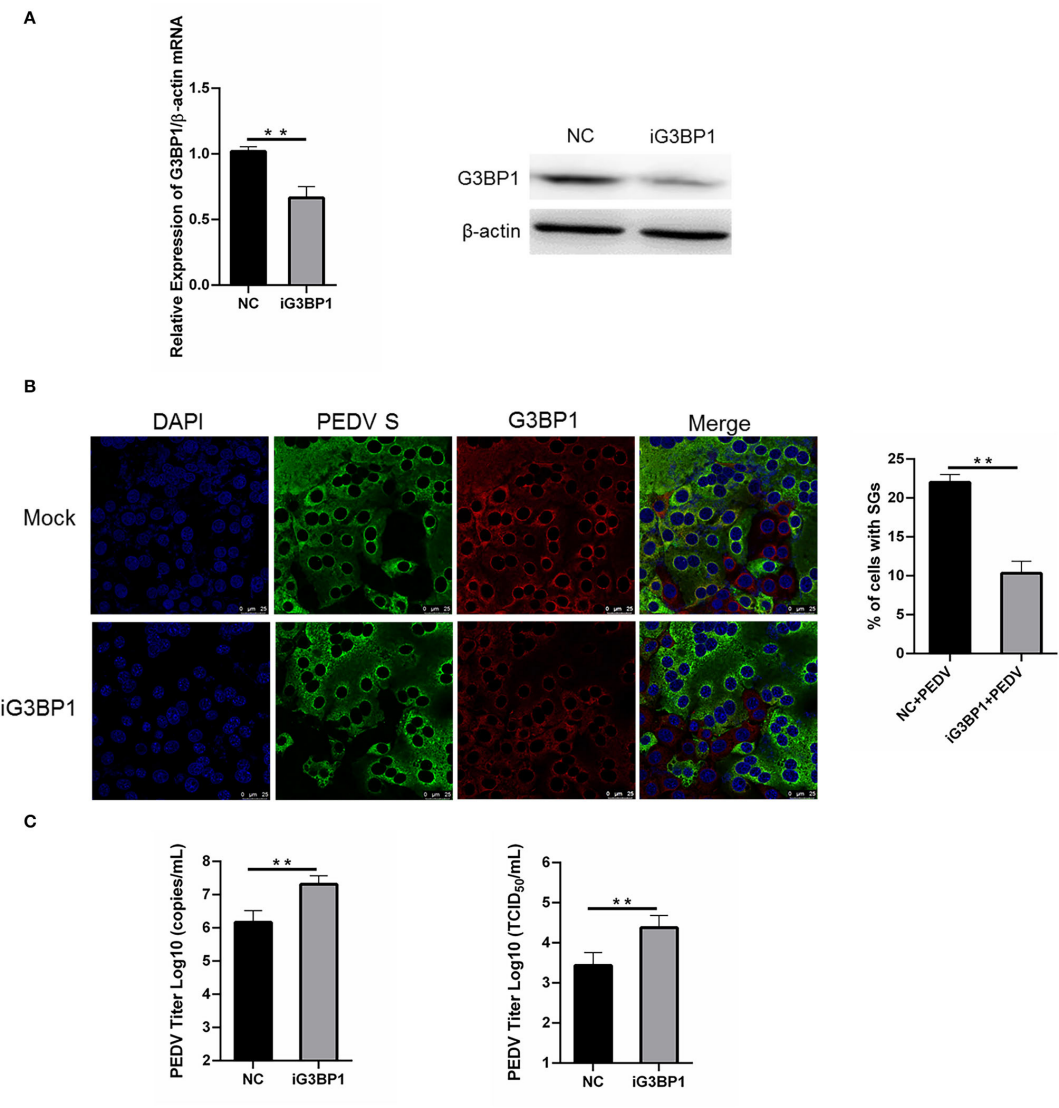
Silencing G3BP1 expression had positive influence on PEDV replication. **(A)** Vero cells were transfected with siRNA G3BP1 or NC for 24 h. The resulting mRNA and protein level of G3BP1 were determined by qRT-PCR and western blot. **(B)** Vero cells were infected with PEDV for another 12 h after transfection with siRNA G3BP1 or NC for 24 h and then the cells were fixed and analyzed by confocal microscopy. Mouse mAb specific for PEDV S was used to detect PEDV infection (green). Rabbit pAb specific for G3BP1 (red) was used to detect SGs formation. Nuclei were stained with DAPI (blue). The percentage of SG-positive cells was quantified as described in the Materials and Methods. The data were presented as mean ± SEM of three independent experiments (*t*-test, **p < 0.01). **(C)** Vero cells were treated as **(B)**. PEDV titer was determined by qRT-PCR and TCID_50_. The data was presented as mean ± SEM of three independent experiments (*t*-test, **p < 0.01).

To assess whether SGs are associated with PEDV replication, we tried to impair SG formation by silencing G3BP1 expression. Specific siRNA was designed, and the knockdown efficiency was demonstrated by the results of real-time RT-qPCR and western blot assays (Figure 5A). The specific siRNA targeting G3BP1 was transfected into Vero cells, followed by PEDV infection. The formation of SGs monitored by immunostaining decreased by 50% in G3BP1-silenced cells compared with NC-treated cells, while the fluorescence signal of PEDV infection was enhanced in G3BP1-silenced cells (Figure 5B). These data indicated that the downregulation of G3BP1 can affect the formation of PEDV-induced SGs. Data from Figure 5C further demonstrated that the suppression of G3BP1 expression obviously increased the viral titer and virus copy number compared to the control group. All the aforementioned data prompted us to draw the conclusion that SGs formation had a negative influence on PEDV replication.

## Discussion

Research on SGs induced by virus infection has rapidly advanced in recent years. Here, we observed that PEDV infection does not induce SGs formation in most infected Vero cells. Meanwhile, the downregulation of G3BP1 enhanced PEDV replication, which was consistent with previous studies (Pandey et al., 2019). It is proposed that SGs exert specific antiviral activities by providing a platform for interaction between antiviral proteins and non-self RNA (Gao et al., 2021). Similar observations were reported for other coronaviruses, including mouse hepatitis virus (MHV), transmissible gastroenteritis virus (TGEV), and severe acute respiratory syndrome coronavirus 2 (SARS-CoV-2) (Raaben et al., 2007; Sola et al., 2011; Cascarina and Ross, 2020). Whether it is a common feature of coronaviruses to modulate SGs requires future works.

Stress granules subversion during virus infection is observed in many viruses. Different viruses disrupt SG-related proteins by various approaches. For example, the foot-and-mouth disease virus (FMDV) L^pro^ cleaves the known SG scaffold proteins G3BP1 and G3BP2 to inhibit SGs formation (Visser et al., 2019). Middle East Respiratory Syndrome Coronavirus (MERS-CoV) can inhibit SGs *via* accessory protein 4a and lead to efficient viral replication (Rabouw et al., 2016; Nakagawa et al., 2018). In this study, we found PEDV-infected cells were resistant to SA-induced SGs formation. Further research indicated that PEDV infection disrupted SGs formation by downregulating G3BP1 expression. It was reported that the activation of the antioxidant pathway by the virus might mediate the inhibition of SGs formation induced by SA treatment since virus infection typically induced both ROS and antioxidant response simultaneously (Basu et al., 2017; Pandey et al., 2019). Although current research reported that PEDV infection induced caspase-8 mediated G3BP1 cleavage and subverted SGs to promote viral replication (Sun et al., 2021), the concrete mechanism of PEDV to circumvent the formation of anti-viral SGs induced by SA treatment needs further investigation.

Previous studies showed that viral protease protein 3C protease (3Cpro) and L protease (Lpro) participated in SGs' disassembly (White et al., 2007; Visser et al., 2019). As is known, PEDV produces two proteases (Nsp3 papain-like protease and Nsp5 3C-like protease) (Wang et al., 2016; Sun et al., 2021). In the present study, we found that PEDV PLP1 or PLP2 which have L protease activities suppressed the SGs formation induced by SA treatment indicated by TIA1 or G3BP1, whereas nsp5 did not show such effect. It was well documented that PEDV impaired SG assembly by targeting G3BP1 *via* the host proteinase caspase-8. In addition, it was reported that coronaviruses antagonized the formation of SGs by nsp15 *via* reducing the viral dsRNA accumulation and sequestering/depleting the critical components of SGs (Gao et al., 2021). Our further research demonstrated that papain-like proteases suppressed SGs formation induced by SA treatment *via* downregulating G3BP1 expression. It is well-known that PEDV papain-like proteases have DUB activity and host interferon antagonistic activity in addition to protease activity (Xing et al., 2013; Chu et al., 2022). It has been reported that ubiquitination is not required for SA-induced SG formation (Gwon and Maxwell, 2021), and Vero cells are interferon-deficient cells (Emmott et al., 2010). Therefore, we speculate that papain-like proteases might inhibit SG formation by exerting its proteolytic function. The elaborate mechanisms of papain-like proteases in suppressing SGs induced by SA treatment are currently under investigation in our laboratory. It will be tremendously valuable to obtain the data about PEDV and SGs interaction from porcine intestinal epithelial cells (IPEC-J2), since Vero cells are not natural host cells for PEDV (Guo et al., 2016).

In conclusion, we provided convincing evidence that PEDV infection does not induce SGs formation in most infected Vero cells. Silencing G3BP1 significantly enhanced PEDV replication. Further research indicated that PEDV might subvert SGs formation *via* downregulating G3BP1 expression dependent on PEDV papain-like proteases. A better understanding of the molecular events involved in the formation of SGs and PEDV infection will undoubtedly help us to elucidate the molecular mechanism of PEDV pathogenesis.

## Data Availability Statement

The raw data supporting the conclusions of this article will be made available by the authors, without undue reservation.

## Author Contributions

BH, ZY, and JW designed the experiments. XG, KejY, ZX, LL, YG, FH, XM, KexY, and YL carried out the experiments. XG and KejY analyzed the data and wrote the paper. XG checked and finalized the manuscript. All authors read and approved the final manuscript.

## Funding

This research was funded by Shandong Provincial Natural Science Foundation Youth Project (ZR2021QC054), Shandong Provincial Modern Agricultural Industry and Technology System (SDAIT-08-01), Great Scientific and Technological Innovation Projects in Shandong Province (2020CXGC010801), the Shandong Key Provincial Research and Development Program (2019GNC106044), the National Natural Science Funds (Nos. 32070178 and 32002286), and Agricultural Scientific and Technological Innovation Project of Shandong Academy of Agricultural Sciences (CXGC2021A12 and CXGC2021A39).

## Conflict of Interest

The authors declare that the research was conducted in the absence of any commercial or financial relationships that could be construed as a potential conflict of interest.

## Publisher's Note

All claims expressed in this article are solely those of the authors and do not necessarily represent those of their affiliated organizations, or those of the publisher, the editors and the reviewers. Any product that may be evaluated in this article, or claim that may be made by its manufacturer, is not guaranteed or endorsed by the publisher.

## References

[B1] Anderson P. Kedersha N. (2009). Stress granules. Curr. Biol. 19, R397–R398. 10.1016/j.cub.2009.03.013 19467203

[B2] Basu M. Courtney S. C. Brinton M. A. (2017). Arsenite-induced stress granule formation is inhibited by elevated levels of reduced glutathione in West Nile virus-infected cells. PLoS Pathog. 13, e1006240. 10.1371/journal.ppat.1006240 28241074 PMC5344523

[B3] Cascarina S. M. Ross E. D. (2020). A proposed role for the SARS-CoV-2 nucleocapsid protein in the formation and regulation of biomolecular condensates. FASEB J. 34, 9832–9842. 10.1096/fj.202001351 32562316 PMC7323129

[B4] Chu H. F. Cheng S. C. Sun C. Y. Chou C. Y. Lin T. H. Chen W. Y. (2022). Structural and biochemical characterization of porcine epidemic diarrhea virus papain-like protease 2. J. Virol. 96, e0137221. 10.1128/jvi.01372-21 34643430 PMC8754210

[B5] Emmott E. Rodgers M. A. Macdonald A. McCrory S. Ajuh P. Hiscox J. A. (2010). Quantitative proteomics using stable isotope labeling with amino acids in cell culture reveals changes in the cytoplasmic, nuclear, and nucleolar proteomes in Vero cells infected with the coronavirus infectious bronchitis virus. Mol. Cell Proteomics 9, 1920–1936. 10.1074/mcp.M900345-MCP200 20467043 PMC2938107

[B6] Gao B. Gong X. Fang S. Weng W. Wang H. (2021). Inhibition of anti-viral stress granule formation by coronavirus endoribonuclease nsp15 ensures efficient virus replication. PLos Pathog. 17, e1008690. 10.1371/journal.ppat.1008690 33635931 PMC7946191

[B7] Guo X. Hu H. Chen F. Li Z. Ye S. Cheng S.. (2016). iTRAQ-based comparative proteomic analysis of Vero cells infected with virulent and CV777 vaccine strain-like strains of porcine epidemic diarrhea virus. J Proteomics 130, 65–75. 10.1016/j.jprot.2015.09.002 26361011 PMC7102838

[B8] Guo X. Zhang M. Zhang X. Tan X. Guo H. Zeng W.. (2017). Porcine epidemic diarrhea virus induces autophagy to benefit its replication. Viruses 9, 53. 10.3390/v9030053 28335505 PMC5371808

[B9] Gwon Y. Maxwell B. A. (2021). Ubiquitination of G3BP1 mediates stress granule disassembly in a context-specific manner. Scienece 372, eabf6548. 10.1126/science.abf6548 34739333 PMC8574224

[B10] Humoud M. N. Doyle N. Royall E. Willcocks M. M. Sorgeloos F. van Kuppeveld F.. (2016). Feline calicivirus infection disrupts assembly of cytoplasmic stress granules and induces G3BP1 cleavage. J. Virol. 90, 6489–6501. 10.1128/jvi.00647-16 27147742 PMC4936126

[B11] Le Sage V. Cinti A. McCarthy S. Amorim R. Rao S. Daino G. L.. (2017). Ebola virus VP35 blocks stress granule assembly. Virology 502, 73–83. 10.1016/j.virol.2016.12.012 28013103

[B12] Lee C. (2015). Porcine epidemic diarrhea virus: an emerging and re-emerging epizootic swine virus. Virol. J. 12, 193. 10.1186/s12985-015-0421-2 26689811 PMC4687282

[B13] Li W. Li H. Liu Y. Pan Y. Deng F. Song Y.. (2012). New variants of porcine epidemic diarrhea virus, China, 2011. Emerg. Infect Dis. 18, 1350–1353. 10.3201/eid1808.120002 22840964 PMC3414035

[B14] Lindquist M. E. Lifland A. W. Utley T. J. Santangelo P. J. Crowe J. E. Jr. (2010). Respiratory syncytial virus induces host RNA stress granules to facilitate viral replication. J. Virol. 84, 12274–12284. 10.1128/jvi.00260-10 20844027 PMC2976418

[B15] Nakagawa K. Narayanan K. Wada M. Makino S. (2018). Inhibition of stress granule formation by middle east respiratory syndrome coronavirus 4a accessory protein facilitates viral translation, leading to efficient virus replication. J. Virol. 92, e00902-18. 10.1128/jvi.00902-18 30068649 PMC6158436

[B16] Niederwerder M. C. Hesse R. A. (2018). Swine enteric coronavirus disease: a review of 4 years with porcine epidemic diarrhoea virus and porcine deltacoronavirus in the United States and Canada. Transbound Emerg. Dis. 65, 660–675. 10.1111/tbed.12823 29392870 PMC7169865

[B17] Onomoto K. Yoneyama M. Fung G. Kato H. Fujita T. (2014). Antiviral innate immunity and stress granule responses. Trends Immunol. 35, 420–428. 10.1016/j.it.2014.07.006 25153707 PMC7185371

[B18] Panas M. D. Ivanov P. Anderson P. (2016). Mechanistic insights into mammalian stress granule dynamics. J. Cell Biol. 215, 313–323. 10.1083/jcb.201609081 27821493 PMC5100297

[B19] Pandey K. Zhong S. Diel D. G. Hou Y. Wang Q. Nelson E.. (2019). GTPase-activating protein-binding protein 1 (G3BP1) plays an antiviral role against porcine epidemic diarrhea virus. Vet. Microbiol. 236, 108392. 10.1016/j.vetmic.2019.108392 31500725 PMC7117524

[B20] Raaben M. Groot Koerkamp M. J. Rottier P. J. de Haan C. A. (2007). Mouse hepatitis coronavirus replication induces host translational shutoff and mRNA decay, with concomitant formation of stress granules and processing bodies. Cell Microbiol. 9, 2218–2229. 10.1111/j.1462-5822.2007.00951.x 17490409 PMC7162177

[B21] Rabouw H. H. Langereis M. A. Knaap R. C. Dalebout T. J. (2016). Middle east respiratory coronavirus accessory protein 4a inhibits PKR-mediated antiviral stress responses. PLoS Pathog. 12, e1005982. 10.1371/journal.ppat.1005982 27783669 PMC5081173

[B22] Sola I. Galán C. Mateos-Gómez P. A. Palacio L. Zúñiga S. Cruz J. L.. (2011). The polypyrimidine tract-binding protein affects coronavirus RNA accumulation levels and relocalizes viral RNAs to novel cytoplasmic domains different from replication-transcription sites. J. Virol. 85, 5136–5149. 10.1128/jvi.00195-11 21411518 PMC3126201

[B23] Sun L. Chen H. Ming X. Bo Z. Shin H. J. Jung Y. S.. (2021). Porcine epidemic diarrhea virus infection induces caspase-8-mediated G3BP1 cleavage and subverts stress granules to promote viral replication. J. Virol. 95, e02344-20. 10.1128/jvi.02344-20 33568512 PMC8104091

[B24] Visser L. J. Medina G. N. Rabouw H. H. de Groot R. J. Langereis M. A. de Los Santos T.. (2019). Foot-and-mouth disease virus leader protease cleaves G3BP1 and G3BP2 and inhibits stress granule formation. J. Virol. 93, e00922-18. 10.1128/jvi.00922-18 30404792 PMC6321903

[B25] Wang D. Fang L. Shi Y. Zhang H. Gao L. Peng G.. (2016). Porcine epidemic diarrhea virus 3C-like protease regulates its interferon antagonism by cleaving NEMO. J. Virol. 90, 2090–2101. 10.1128/jvi.02514-15 26656704 PMC4733996

[B26] White J. P. Cardenas A. M. Marissen W. E. Lloyd R. E. (2007). Inhibition of cytoplasmic mRNA stress granule formation by a viral proteinase. Cell Host Microbe 2, 295–305. 10.1016/j.chom.2007.08.006 18005751

[B27] Xing Y. Chen J. Tu J. Zhang B. Chen X. Shi H.. (2013). The papain-like protease of porcine epidemic diarrhea virus negatively regulates type I interferon pathway by acting as a viral deubiquitinase. J. Gen. Virol. 94(Pt 7), 1554–1567. 10.1099/vir.0.051169-0 23596270 PMC4811637

[B28] Yoneyama M. Jogi M. Onomoto K. (2016). Regulation of antiviral innate immune signaling by stress-induced RNA granules. J. Biochem. 159, 279–286. 10.1093/jb/mvv122 26748340 PMC4763080

[B29] Zhou Y. Fang L. Wang D. Cai K. Chen H. Xiao S. (2017). Porcine reproductive and respiratory syndrome virus infection induces stress granule formation depending on protein kinase R-like endoplasmic reticulum kinase (PERK) in MARC-145 cells. Front. Cell Infect. Microbiol. 7, 111. 10.3389/fcimb.2017.00111 28421170 PMC5378712

